# The establishment of a multiplex fluorescent polymerase chain reaction coupled with capillary electrophoresis analysis technology enables the simultaneous detection of 16 genotypes of human papillomavirus

**DOI:** 10.1002/jcla.24996

**Published:** 2023-12-22

**Authors:** Lingling Nie, Haiyang Qin, Sisi Li, Zailiang Yu, Weijin Huang, Li Zhang, Jian Zhao

**Affiliations:** ^1^ Division of HIV/AIDS and Sex‐transmitted Virus Vaccines National Institutes for Food and Drug Control Beijing China; ^2^ Suzhou YueMicro Gene Technology Co. Ltd Suzhou China; ^3^ Department of Obstetrics and Gynecology Peking University First Hospital Beijing China

**Keywords:** capillary electrophoresis, cervical cancer screening, gene analysis, HPV, multiplex fluorescent PCR

## Abstract

**Background:**

The detection and accurate genotyping of human papillomavirus (HPV) infection is critical for preventing and effectively treating cervical cancer.

**Methods:**

A multiplex fluorescent polymerase chain reaction (PCR) coupled with a capillary electrophoresis method was developed for the simultaneous detection of the 16 most prevalent HPV genotypes. Twenty‐five pairs of primers were ultimately selected to ensure that both E and L regions of nine HPV genotypes, as well as the E regions of seven HPV genotypes could be accurately amplified.

**Results:**

This method enables the simultaneous detection and differentiation of 16 HPV genotypes in a single closed‐tube reaction, accurately distinguishing products with molecular weight differences >1 bp through capillary electrophoresis. This method demonstrated exceptional accuracy, specificity, and repeatability with a detection limit of 10 copies/μL for all 16 HPV genotypes. Furthermore, 152 cervical swab specimens were obtained to compare the disparities between this approach and Cobas 4800 HPV detection method. The concordance rate and *κ* value were 90.1% and 0.802, respectively, indicating a high level of agreement. The established detection method was successfully applied to cervical swab specimens for determining HPV genotypes across all levels of cervical lesions, HPV52, 56, 16, and 59 were found to be most prevalent with infection rates of 10.8%, 9.1%, 6.5%, and 6.2%, respectively.

**Conclusions:**

This study has successfully established a detection method capable of simultaneously identifying 16 HPV genotypes. This approach can be further applied to HPV vaccine research and surveillance, with the potential for broad applications.

## Graphical abstract

A multiplex fluorescent polymerase chain reaction coupled with a capillary electrophoresis method was developed for the simultaneous detection of the 16 most prevalent human papillomavirus (HPV) genotypes. Twenty‐five pairs of primers were ultimately selected to ensure that both E and L regions of nine HPV genotypes, as well as the E regions of seven HPV genotypes could be accurately amplified.

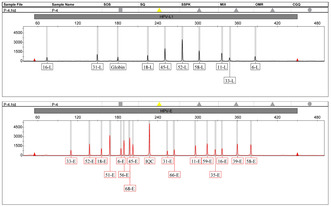

## 1 INTRODUCTION

Cervical cancer is one of the major diseases that critically affects women's health.^1^ Human papillomavirus (HPV) infection, especially persistent HPV infection, is the main factor in cervical cancer and precancerous lesions.^2^, ^3^ HPV genotypes are classified into high‐risk (HR), middle‐risk, and low‐risk (LR) groups based on their pathogenicity.^4^ High‐risk HPV has been detected in more than 99% of cervical cancers worldwide,^5^ while low‐risk HPV genotypes, such as HPV6 and 11, contribute to the development of genital warts.^6^ The World Health Organization's “Guidelines for Screening and Treatment of Cervical Pre‐Cancer Lesions for Cervical Cancer Prevention” (2nd edition) recommend that HR‐HPV detection be used as the primary screening technique for cervical cancer, as it is superior to traditional cytology and acetic acid/iodine staining in terms of visual observation screening.^7^ Therefore, the early screening and accurate typing of HPV are crucial for timely diagnosis, prognosis prediction and post‐treatment monitoring of cervical cancer. Moreover, numerous Chinese companies have invested in the research and development of multivalent cervical cancer vaccines. The majority of these companies are focusing on 9‐valent vaccines (HPV6, 11, 16, 18, 31, 33, 45, 52, and 58), while some are working on higher valent (i.e., 14‐valent and 15‐valent) that primarily target HPV6, 11, 16, 18, 31, 33, 35, 39, 45, 51, 52, 56, 58, 59, 66, and 68.^8^ In clinical trials for HPV vaccines, the accuracy of HPV genotyping is crucial to evaluate the protective effect of vaccination. Thus, it is imperative to establish a precise genotyping method that specifically targets HPV6, 11 as well as the high‐risk genotypes (16, 18, 31, 33, 35, 39, 45, 51, 52, 56, 58, 59, 66, and 68).

According to statistics, by 2020, there were over 425 HPV genotyping detection kits available on the global market utilizing various technologies such as real‐time polymerase chain reaction (PCR), Luminex, microarrays, and DNA hybridization.^9^ Despite the increasing availability of commercial HPV tests over the past decade, a staggering 82% of these tests lack any published analytical or clinical evaluation.^9^ Currently, fluorescent quantitative PCR is a widely utilized method for HPV genotype detection due to its high sensitivity, automation capabilities, and cost‐effectiveness. Moreover, the operation is straightforward and detection can be achieved in a single step within a closed tube. However, this method demands meticulous primer and probe design, exhibits high false‐positive rates, and has limited typing capacity. The Cobas 4800 HPV DNA detection kit has undergone clinically validation and received approval from the U.S. Food and Drug Administration for its use in primary screening of cervical cancer.^10^, ^11^ The Cobas HPV test exhibits high clinical sensitivity and negative predictive value in the identification of high‐grade cytopathies.^10^ The Cobas HPV test is capable of detecting DNA from HPV16, 18, 31, 33, 35, 39, 45, 51, 52, 56, 58, 59, 66, and 68 DNA with the ability to differentiate between types 16 and 18. The cycle threshold cut‐off values for the Cobas HPV test have been established through analysis of samples from approximately 29,000 women in the ATHENA clinical trial.^12^ Therefore, a new HPV DNA genotyping method can be evaluated by comparing its clinical sample detection accuracy with that of the Cobas HPV detection test.

In this study, a novel multiplex fluorescence PCR method was developed for the simultaneous detection of 16 HPV types (HPV6, 11, 16, 18, 31, 33, 35, 39, 45, 51, 52, 56, 58, 59, 66, and 68) by consistently refining the design of primers and probes. The resulting PCR products were subsequently analyzed using capillary electrophoresis technology to achieve precise genotype differentiation. Additionally, collection of clinical samples enabled comparison between this method and Cobas 4800 HPV detection test to evaluate its efficacy. Finally, additional clinical samples were collected to assess the prevalence of HPV genotypes across all grades of cervical lesions.

## 2 MATERIALS AND METHODS

### 2.1 Reference materials and clinical samples

In this study, the national reference substance for whole‐genome HPV genotyping (batch number 360003‐201602) was utilized, comprising of 22 HPV genotype substances (a concentration of approximately 106–107 copies/mL) and five negative reference substances (HSV2, CMV, UU, CT, and human genome). National standards for HPV51 and 56 types were not included in this purchased batch of national standards. Therefore, plasmids containing HPV51 and 56 in zone L and E synthesized by Shanghai Jierui Bioengineering Co., Ltd. were utilized for the performance verification of the kit in this study.

From June to September 2020, 379 cervical swab specimens with corresponding cell histopathological information were prospectively collected from women who underwent cervical fluid‐based cytology test at the gynecological outpatient department of Peking University First Hospital. The samples were initially preserved in Thinprep® Pap Test PreservCyt® cell preservation solution and subsequently stored at −80°C for further analysis. Normal cytology was observed in 198 cases, while abnormal cytology was found in 181 cases. Histopathology examination revealed mild inflammation in 28 cases, clue cells in 13 cases, fungal infection in two cases, epidermal cell atrophy in 22 cases, mild inflammation combined with epidermal cell atrophy in one case, atypical squamous cell changes in 99 cases, low‐grade squamous intraepithelial cytopathy in 14 cases, and high‐grade squamous intraepithelial cell lesions in two cases. Among the samples, 157 cervical swab specimens with patient information were simultaneously analyzed in the hospital using the Cobas 4800 HPV detection method according to the manufacturer's instruction, and the genotyping results of the sample were obtained.

Of the 379 samples subjected to the established methodology, five failed to yield valid outcomes due to DNA extraction issues, one was mislabeled, and another was suspected of contamination. Consequently, a total of 372 samples were available for analysis.

### 2.2 Instruments and reagents

The GeneAmp® 9700 PCR system and the ABI 3730 Genetic Analyzer were procured from Applied Biosystems, while nucleic acid extraction was performed using the QIAamp DNA mini‐Kit. Microreader™ Master Mix I (Suzhou Microread Genetics Co., Ltd, Suzhou, China) was utilized in the PCR reaction.

The 16‐valent human papillomavirus DNA detection kit was independently developed. The primer pairs were mainly designed primarily targeting various types of HPV L1 and E regions, and synthesized by Shanghai Jierui Bioengineering Co., Ltd. This kit enables simultaneous detection of 16 common HPV genotypes (HPV6, 11, 16, 18, 31, 33, 35, 39, 45, 51, 52, 56, 58, 59, 66, and 68).

### 2.3 The establishment and optimization of a multiplex fluorescent PCR‐based HPV nucleic acid genotyping detection method

To investigate the feasibility of simultaneous amplification of multiple target fragments with high specificity and sensitivity using multiplex PCR, we optimized the primers for multiplex PCR amplification by designing them based on the early (E) and late (L) gene regions of HPV, and generating multiple pairs of primers for each HPV genotype. The primer pairs exhibiting optimal specificity were ultimately selected. The 5′ end of one of the specific primer pairs was fluorescently labeled to ensure efficient amplification of all 16 HPV genotypes. The amplification of various genotypes resulted in PCR products with distinct fluorescent markers and fragment lengths, enabling accurate differentiation of each genotype. The PCR reaction protocol was conducted as follows: initial denaturation at 95°C for 5 min, followed by 27–32 cycles of amplification consisting of denaturation at 94°C for 20 s and annealing at 61°C for 90 s; and a final extension step at 60°C lasting between 30 and 60 min. Optimal concentrations of enzymes, primers, and probes were determined through detection of national reference samples containing HPV genotypes.

The PCR products were resolved by electrophoresis using an ABI 3730 genetic analyzer. The capillary with a length of 50 cm was filled with poly‐dimethylacrylamide POP‐7 as the polymer separation matrix, and the running buffer used was 3730 running buffer (Applied Biosystems™). The electrophoresis procedure involved adding 8.5 μL of deionized Hi‐Di formamide and 0.5 μL of molecular weight marker to 1 μL PCR product, followed by thorough mixing. Subsequently, the samples were loaded with 1.5 kV for a duration 8 s and then subjected to electrophoresis at a voltage of 15 kV for 50 min. Upon completion of the electrophoretic detection process, data analysis was conducted using Gene Mapper v 5.0 software with a lower detection limit for peak height detection set at 200 RFU.

### 2.4 Evaluation of the accuracy of HPV nucleic acid genotyping using multiplex fluorescent PCR

The national references for whole genotyping of HPV6, 11, 16, 18, 31, 33, 35, 39, 45, 51, 52, 56, 58, 59, 66, and 68 were diluted to a concentration of 100 copies/μL. These diluted samples of the national reference materials were utilized as templates in accordance with the established method outlined in Section 2.3. Subsequently, the accuracy of this methodology was evaluated.

### 2.5 Evaluation of specificity, reproducibility, and sensitivity of HPV nucleic acid genotyping using multiplex fluorescent PCR

The HPV26, 61, 69, 71, 73, 81, and 82 national reference samples were diluted to a concentration of 100 copies/μL and utilized as templates for assessing the method specificity. Concurrently, five negative reference products (HSV2, CMV, UU, CT, and human genome) lacking HPV genotypes were examined to further evaluate the method's specificity.

The HPV6 and 11 national reference samples for whole genotyping were diluted to a concentration of 100 copies/μL. To assess the method's repeatability, the diluted samples were utilized as templates and subjected to 10 repeated assays using the optimized PCR reaction system and conditions.

National reference samples of HPV6, 11, 16, 18, 31, 33, 35, 39, 45, 52, 58, 59, 66, and 68 were subjected to serial dilution at concentrations of 100, 50, 10, and 1 copy/μL. These diluted samples were utilized as templates for assessing the sensitivity of the established method.

### 2.6 Statistical analysis

To assess the detection performance of this method, detection results were compared with those of the Cobas 4800 HPV detection method adopted by the hospital and the level of agreement was analyzed by Cohen's kappa statistics. A *κ* value exceeding 0.75 indicates excellent agreement. The statistical analyses were conducted using SPSS 21 software.

## 3 RESULTS

### 3.1 Established HPV nucleic acid genotyping detection method based on multiplex fluorescent PCR combined with capillary electrophoresis analysis technology

Multiple pairs of amplification primers were designed for each fragment based on the E and L gene regions of HPV. After careful selection, only Primers with high efficiency and specificity were chosen for multiple amplification. The primers that may have exhibited non‐specific were redesigned based on multiple amplification results until the system specificity and efficiency met the required standards. Ultimately, a total of 28 pairs of primers were designed and listed in Table 1. Among them, a set of 25 primers pairs were carefully selected to ensure efficient amplification of the E and L regions of 9 HPV genotypes (HPV6, 11, 16, 18, 31, 33, 45, 52, and 58), as well as the E regions of HPV35, 39, 51, 56, 59, 66, and 68. Two pairs were specifically used for amplifying the internal reference gene globin while one pair was used for amplifying Internal Quality Control (IQC). The two pairs of globin DNA served as a positive control to access the entire process encompassing sample collection, DNA extraction, and PCR reaction. The IQC primer amplified synthetic spike DNA targets that were included in the primer mix. The IQC system was implemented as a positive control for the PCR reaction. The PCR reaction system was optimized by adding 4 μL of 2.5× Microreader™ Master Mix I, 2 μL of HPV Primer Mix, 1–4 μL template, and ddH_2_O to make up the volume to 10 μL. These PCR products were separated by electrophoresis using an ABI 3730 Genetic Analyzer that accurately distinguishes products with a molecular weight difference greater than or equal to 1 base pair.

**TABLE 1:** The sequence information of the 28 pairs of designed primers, primer‐labeled dyes, and corresponding amplified fragment sizes.

Primer design Sites	Primer sequence (labeled)	Primer sequence (unlabeled)	Primer‐labeled dyes	Sizes (bp)
6‐L	GATGTTGAAAATTCAGGGAGTG	TGGCAAACATTTGTTCCTTC	TAMRA	387
11‐L	GGTAAGGGTACACAATGTTCAAAT	TATTACCCCCTTTTACCAACAGGT	TAMRA	337
16‐L	GTTGTAAGCACGGATGAATATGT	GTCCAACTGCAAGTAGTCTGGATG	TAMRA	73
18‐L	TGCTTACGGCGTGAGCAG	GAAACACCATTGTTATGACCCT	TAMRA	225
31‐L	ATTCCATACCTAAATCTGACAATC	CAAACTAAGCGTTGAGTTTCAGG	TAMRA	150
33‐L	TCAATGGTTACTTCCGAATCT	GTGTAAACTAGCAGATGGAGGA	TAMRA	349
45‐L	CGCAGGATGTTAGGGATAATG	ATGTCTAATGGAACCTCGCACT	TAMRA	251
52‐L	AAAAACACCAGTAGTGGTAAT	ATCTATACCAGGTTTACCAGC	TAMRA	277
58‐L	GACATGGTAGATACAGGGTTT	AATAGAGCCACTAGGAGTTGGA	TAMRA	303
6‐E	TCTTGTCCGTCCACTTCGTCC	GCGGTTCATAAAGCTAAATTGTA	ROX	184
11‐E	GCCCAGCAAAAGGTCTTGTAG	CTAAATAACCAGTGGAAGGGTCGT	ROX	296
16‐E	TAACAGGTCTTCCAAAGTACGAA	AAGATTCCATAATATAAGGGGTC	ROX	336
18‐E	TCTGAGTCGCTTAATTGCTCGTG	GGAGCACGACAGGAACGACT	ROX	166
31‐E	ACTTACACTGACAACAAAAGGTA	TAGGAGGAAGGTGGACAGGACG	ROX	254
33‐E	GCAGTATAGGTCAGTTGGTTCAGG	GGTCCCGACGTAGAGAAACTGC	ROX	110
35‐E	CTGAACGACCTTACAAACTGCA	GTTTTCAACTGGACACAGCGGT	ROX	327
39‐E	GCTATACAGGACAGTGTCGACG	AGCTGCTGTAGTTGTCGCAGAG	ROX	359
45‐E	CGTAACACAACAGGTCAACAGGA	ACAAACGAAGATTTCACAGCATA	ROX	202
51‐E	GTCACGCATATTATCTACTTCATCC	AACGTACACGACAACGTAACGAA	ROX	169
52‐E	CACCATCTGTATCCTCCTCATCTG	GACCTGTGACCCAAGTGTAACG	ROX	137
56‐E	ATGTTTGGGGTGCTGGAGAC	GCTCCTGCAAATGGTCTACTTC	ROX	189
58‐E	TAGCGTTGGGTTGTTTCCTCTC	ACTTTGCAGCGATCTGAGGTATA	ROX	380
59‐E	ACACCTAAGACAGCAACGACAAG	ATCCGTCTTGCGAGGTTTCTACTAC	ROX	314
66‐E	GCAGTGTTGGAGACATACGAGTAG	GACCACCAACTCACACTTACAACA	ROX	264
68‐E	TCTGCGGTCCTCTCGCTTA	GGGTGTATGCAACTACATTAGAAAC	ROX	199
Globin^1^	CAGGTACGGCTGTCATCACTTAG	AATGGTGTCTGTTTGAGGTTGCTA	TAMRA	180
Globin^2^	TGTCCTTGGCTCTTCTGGCAC	ACTCTTCCACTTTTAGTGCATCAAC	ROX	230
IQC	AAGCTGAGCTGGGCAGAATT	ACTGGTAAAGGTGGGATAAAAGGT	ROX	227

*Note*: 6‐L–58‐L were the primers designed for each fragment based on the L gene regions of HPV. 6‐E–58‐E were the primers designed for each fragment based on the E gene regions of HPV. Globin^1^ and Globin^2^ were the primers designed based on globin gene region. The IQC was the primers designed based on the synthetic spike DNA targets that were included in the primer mix.

### 3.2 The accuracy of HPV nucleic acid genotyping detection based on multiplex fluorescent PCR combined with capillary electrophoresis analysis

**FIGURE 1:**
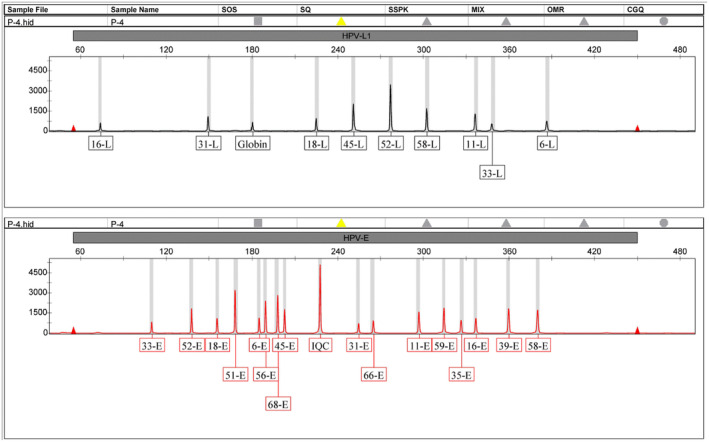
The amplification results of 27 pairs of primers for detecting the mixed 16 national reference materials. 6‐L, 11‐L, 16‐L, 18‐L, 31‐L, 33‐L, 45‐L, 52‐L, and 58‐L represent amplification results of the L‐region of the corresponding genotype, 6‐E, 11‐E, 16‐E, 18‐E, 31‐E, 33‐E, 35‐E, 39‐E, 45‐E, 51‐E, 52‐E, 56‐E, 58‐E, 59‐E, 66‐E, and 68‐E represent amplification results of the E‐region of the corresponding genotype. Globin represents the amplification results of Globin^1^, IQC represent the amplification results of internal quality control.

Positive amplification signals were detected for all diluted national reference materials used in the whole genotyping of HPV6, 11, 16, 18, 31, 33, 35, 39, 45, 51, 52, 56, 58, 59, 66, and 68. Additionally, a sample was prepared by mixing diluted national reference materials of HPV genotypes HPV6, 11, 16, 18, 31, 33, 35, 39, 45, 51, 52, 56, 58, 59, 66, and 68 to evaluate the detection accuracy of the method. The results demonstrated that the 27 designed primer sequences were simultaneous efficient amplified and accurately distinguished by capillary electrophoresis (Figure 1).

### 3.3 The specificity, reproducibility, and sensitivity of HPV DNA genotyping detection based on the established method

**FIGURE 2:**
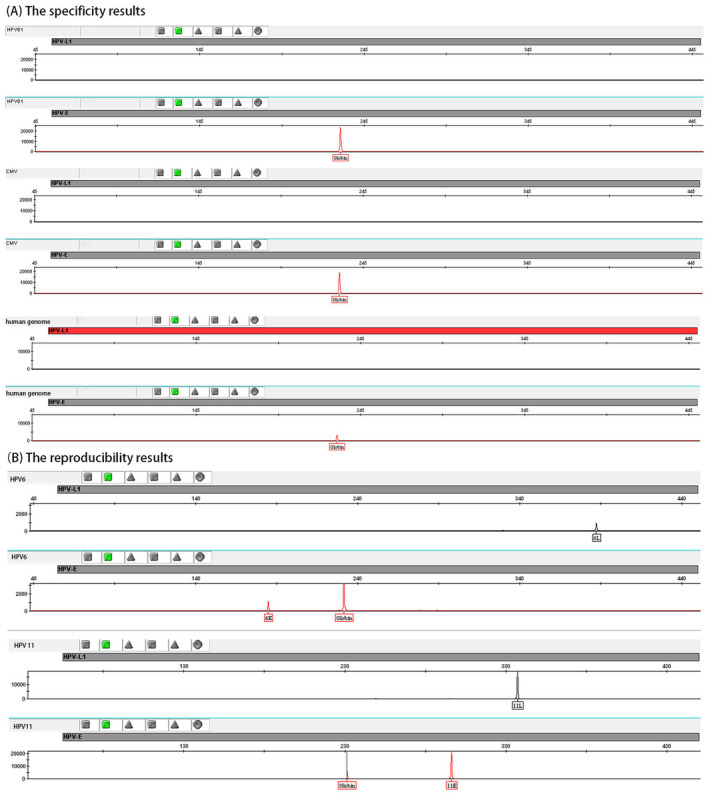
The representative specificity and reproducibility results of this method. (A) The amplification results of 26 pairs of primers for detecting complete genotypes of HPV81, negative reference samples free of HPV genotypes (CMV), and human genome. (B) The amplification results of 26 pairs of primers for detecting complete genotypes of HPV6 and 11. HPV L1 and HPVE represent the amplification results in the two fluorescent channels. 6L and 11L represent a repetitive amplification result of the L‐region of the corresponding genotype. 6E and 11E, represent a repetitive amplification result of the E‐region of the corresponding genotype. Globin represents the amplification results of Globin^2^.

To evaluate the specificity of the established method, we selected diluents from national reference samples that contain complete genotypes of HPV26, 61, 67, 69, 71, 73, 81, and 82 as well as five negative reference samples free of HPV genotypes (HSV2, CMV, UU, CT, and human genome) for detection. No positive results were detected in any sample tested using the established method, the representative specificity results of this method were shown in Figure 2A, thus, indicating its excellent specificity.

Diluents (100 copies/μL) of national reference samples of HPV6 and 11 underwent 10 repeated tests, consistently yielding positive outcomes for the corresponding HPV genotypes with a repetition rate of 100%, and the representative reproducibility results of this method are shown in Figure 2B. These results confirm the excellent detection repeatability.

**FIGURE 3:**
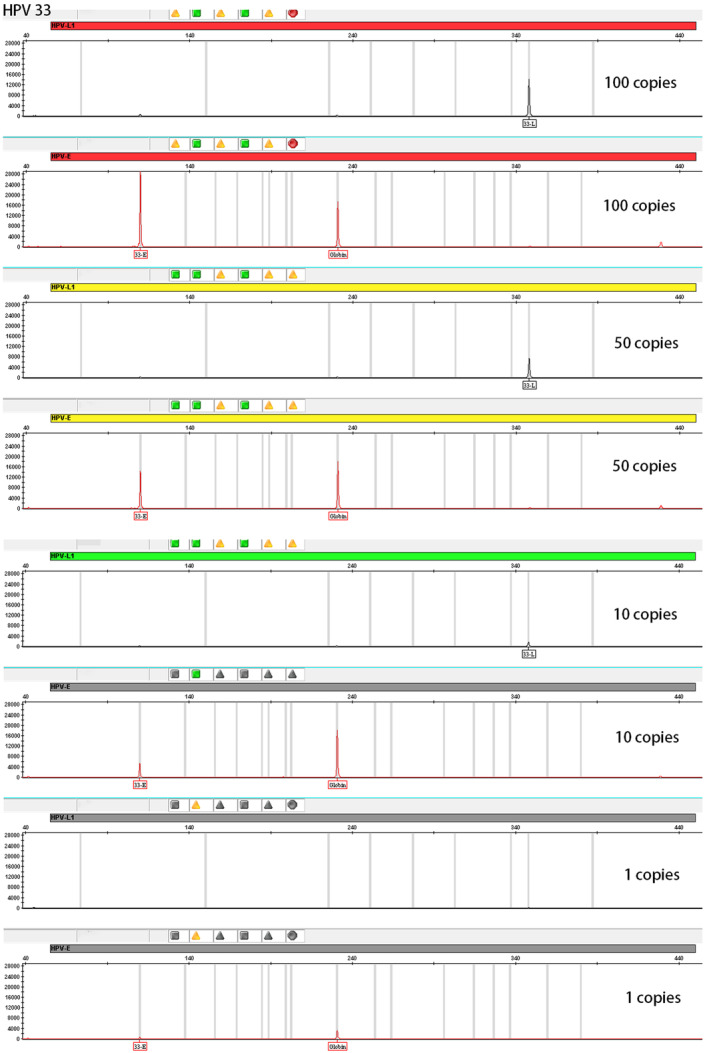
The representative sensitivity results of this method. The amplification results of 26 pairs of primers for detecting complete genotypes of HPV33 at the concentration of 100, 50, 10, and 1 copies/μL. HPV L1 and HPV E represent the amplification results in the two fluorescent channels. Globin represents the amplification results of Globin^2^.

The sensitivity of the HPV nucleic acid typing method based on capillary electrophoresis was evaluated by diluting national reference samples to 100, 50, and 10 copies/μL of 14 HPV genotypes. The detection rates were found to be 100% at all three dilutions. However, only eight out of the 14 HPV types could be detected in the national reference dilution solution at 1 copy/μL, the representative sensitivity results of this method of HPV33 were shown in Figure 3. Therefore, following industry standard, the minimum detection limit of this technique was determined to be 10 copies/reaction.

### 3.4 Concordance between the established method and the Cobas HPV 4800 assay for the detection of HPV DNA

A total of 157 cervical swab specimens were simultaneously analyzed using the established method and Cobas HPV 4800 assay. Among them, 152 validated results used to compare the consistency between the two methods. To ensure a more accurate comparison, the results obtained from the established method were converted as the three categories of Cobas HPV 4800 to align with those of the Cobas HPV 4800 test. The three categories are as follows: HPV16, 18 and 12 high‐risk HPV genotypes (HPV31, 33, 35, 39, 45, 51, 52, 56, 58, 59, 66, and 68). HPV6 and 11 detected by the established method were excluded from this analysis. The comparison results of 152 samples detected by Cobas HPV 4800 assay and the established methods are presented in Table 2. The concordance rates of HPV16, 18 and 12 high‐risk HPV genotypes were 100%, 97.3%, and 88.8%, respectively and the corresponding *κ* value were 1, 0.490 (95% CI: 0.067–0.913), and 0.774 (95% CI: 0.674‐0.874), respectively. The results showed perfect agreement for HPV16, while the determination of agreement for HPV18 was challenging due to a limited number of positive specimens resulting in a 95% confidence interval of 0.067–0.913. The 12 high‐risk HPV genotypes had values >0.75, indicating good agreement between the two methods. Overall, the concordance rate between positive and negative results for all 14 HPV genotypes was determined to be 90.1%, with a *κ* value of 0.803 indicating substantial agreement.

**TABLE 2:** Comparison results of the 152 samples detected by the Cobas established methods and the established methods.

Genotypes	No. of samples with Cobas HPV 4800 and the established method				Concordance rate (%)	*κ* value	95% CI
	+/+	−/−	+/−	−/+			
HPV16	14	138	0	0	100	1	1–1
HPV18	2	146	0	4	97.3	0.490	0.067–0.913
12 high‐risk HPV	59	76	4	13	88.8	0.774	0.674‐0.874
Positive	74	63	3	12	90.1	0.802	0.707–0.896

*Note*: +/+, −/−, +/−, −/+, the former set represents the results obtained by Cobas HPV 4800, the latter set represents those obtained the established method.

Abbreviations: CI, confidence interval; HPV, human papillomavirus.

### 3.5 The occurrence of HPV genotypes in all levels of cervical lesions

The established method was employed to analyze the occurrence of HPV genotypes across all levels of cervical lesions in 372 samples. A total of 150 cases of HPV infection were detected, with a positive rate of 40.3%. Among them, there were 112 cases of single‐type infection cases and 38 cases of multiple infection. Among the cases of multiple infections, there were 29 cases of double infection, eight cases of triple infection, and one case of quintuple infection were observed. The genotypes with the highest infection rate were HPV52 (10.8%), followed by HPV56 (9.1%), HPV16 (6.5%), and HPV59 (6.2%; Table 3). Based on the results of Thinprep cytologic testing, 258 cases were classified as negative for intraepithelial lesions (NILM), 99 cases as atypical squamous cells of undetermined significance (ASCUS), 14 cases as low squamous intraepithelial lesions (LSIL), and two cases as high squamous intraepithelial lesions (HSIL). The NILM group was further divided into normal (N, 191 cases), mild inflammation (G1, 28 cases), cue cells (X1, 13 cases), cell atrophy (PM, 22 cases), fungal infection (FI, 2 cases), and mild inflammation combined with cell atrophy (G1&PM, one case; Table 3). In the cervical lesions groups (NILM, ASCUS, LISL), the top four genotypes of infection remained HPV52, 56, 16, and 59 (Table 3).

**TABLE 3:** The occurrence of HPV genotypes in all levels of cervical lesions.

HPV genotypes	NILM							ASCUS (*n* = 99)	LSIL (*n* = 14)	HISL (*n* = 2)	Total (*n* = 372)
	Normal (*n* = 191)	G1 (*n* = 28)	X1 (*n* = 13)	FI (*n* = 2)	PM (*n* = 22)	G1&PM (*n* = 1)	Total (*n* = 258)				
Negative	116	16	6	2	10	0	150	67	4	1	222 (59.7%)
6	1	0	0	0	0	0	1	0	0	0	1 (0.2%)
16	16	1	0	0	2	0	19	4	1	0	24 (6.5%)
18	3	1	2	0	1	0	7	0	0	0	7 (1.9%)
31	4	0	1	0	0	0	5	1	1	0	7 (1.9%)
33	3	1	0	0	1	0	5	1	1	1	8 (2.2%)
35	1	0	0	0	0	0	1	0	0	0	1 (0.2%)
39	4	2	1	0	1	0	8	2	1	0	11 (3.0%)
45	0	0	0	0	1	0	1	0	0	0	1 (0.2%)
51	8	2	0	0	0	0	10	3	1	0	14 (3.8%)
52	14	5	2	0	3	0	24	12	4	0	40 (10.8%)
56	17	3	1	0	2	0	23	9	2	0	34 (9.1%)
58	11	2	0	0	1	1	15	2	0	0	17 (4.5%)
59	16	0	1	0	1	0	18	4	1	0	23 (6.2%)
66	3	0	1	0	1	0	5	2	0	0	7 (1.9%)
68	3	0	0	0	0	0	3	1	0	0	4 (1.1%)
Total	220	33	15	2	24	1	295	108	16	2	421

*Notes*: The total number of genotypes in the final count exceeded the total number of samples due to the presence of multiple infections in some samples.

Abbreviations: ASCUS, atypical squamous cells of undetermined significance; FI, fungal infection; G1&PM, mild inflammation combined with cell atrophy; G1, mild inflammation; HSIL, the high squamous intraepithelial lesion; LSIL, low squamous intraepithelial lesion; N, Normal; NILM, no intraepithelial lesions; PM, cell atrophy; X1, cue cells.

## 4 DISCUSSION

Accurate HPV genotyping is essential for facilitating epidemiologic studies, vaccine trials, and HPV‐related cancer research.^13^, ^14^ There are 14 high‐risk HPV types known to cause cervical cancer. We designed and implemented multiplex fluorescent PCR coupled with capillary electrophoresis analysis, a novel tool for accurate HPV detection and genotyping. Initially, this methodology was developed specifically for genotyping clinical samples in HPV vaccine clinical trials, targeting the nine genotypes covered by most 9‐valent vaccines (6, 11, 16, 18, 31, 33, 45, 52, and 58). To ensure accurate detection of low‐titer samples near the threshold level, both L and E gene regions were simultaneously analyzed. However, as some vaccines under development include a broader range of genotypes such as the 14‐valent and 15‐valent vaccines; assessing the clinical effectiveness of these vaccines requires not only detecting infections caused by HPV genotypes targeted by the vaccines, but also excluding them as potential causes if a sample develops disease. Therefore, it is necessary to develop kits that can test for both vaccine‐related HPV genotypes and 14 high‐risk genotypes. At a later stage of development, we expanded the typing capability of the kit to encompass all these additional seven high‐risk genotypes. Nevertheless, the limited specificity of the L‐region in HPV makes it challenging to design 16 specific primers their respective genotypes in a single reaction. As a result, the detection was confined solely to early (E) gene regions of HPV for the remaining seven genotypes (HPV35, 39, 51, 56, 59, 66, and 68). Ultimately, a set of 25 pairs of primers were chosen to enable efficient simultaneous amplification of E and L regions of nine HPV genotypes (HPV6, 11, 16, 18, 31, 33, 45, 52, and 58) as well as the E regions of seven HPV genotypes (HPV35, 39, 51, 56, 59, 66, and 68). Although there are numerous kits available both domestically and internationally, there is a scarcity of typing kits specifically designed to cater particular vaccine coverage types. For instance, the validated HPV Cobas 4800 platform demonstrates its capability in detecting DNA from HPV16, 18, 31, 33, 35, 39, 45, 51, 52, 56, 58, 59, 66, and 68 DNA with the ability to differentiate between types 16 and 18. However, this method is limited as it does not cover genotypes 6 and 11, nor can it genotype the 12 high‐risk genotypes. Additionally, various other detections exist that encompass relevant vaccine genotypes; however, the majority of common PCR‐based kits on the market detection results through color rendering or fluorescence intensity. However, due to the high sensitivity of PCR method, there is a possibility of amplification for aerosols in laboratory environments, leading to a higher false‐positive rate. In contrast, this method utilizes capillary electrophoresis (PCR‐CE) to detect the amplification products with a resolution as fine as 1 bp and differentiates different virus genotypes using specifically designed primers on capillary electrophoresis platforms. Even if there is a non‐specific amplification, they can be excluded at result analysis stage effectively reducing false positives. The performance of this method was evaluated in compliance with the “Guidelines for Human Papillomavirus (HPV) Nucleic Acid Detection, Genotyping, and Reagent Technical Review”.^15^ The results demonstrate the robust analytical performance of this method, which generates reproducible and reliable outcomes. The findings indicated the 16 HPV genotyping detection reagents meet the required product performance index and guidelines in terms of accuracy, specificity, repeatability, and detection limit. Highly sensitive and accurate HPV genotype detection of the 16 HPV genotypes may have major clinical significance and can help identify the risk of developing high‐grade cytopathies. Moreover, type‐specific HPV methods are valuable for evaluating the effectiveness of HPV vaccines, improving the risk stratification of HPV patients in cervical screening programs, and enabling the extension of screening intervals.^16^

Moreover, the established method exhibited good concordance with the Cobas HPV 4800 assay, particularly for HPV16. The established method detected all HPV16 and 18 samples detected by the Cobas test. However, four samples of HPV18 genotypes were detected with the established method but not with the Cobas method. Moreover, the detection rate for 12 HR‐HPV types was higher with the established method than that with the Cobas method. This may be related to the differing sensitivities of the two methods. Several studies that have compared the Cobas 4800 HPV Detection Reagent with the Linear Array HPV genotyping test indicate a high agreement rate. No statistical difference was observed between the two methods in the detection of HPV16 and 18. However, these methods further detected a higher number of other 12 HR‐HPV positives.^10^, ^17^

Finally, the occurrence of HPV genotypes in all levels of cervical lesions was calculated. The most common HR‐HPV genotypes were HPV52, 56, 16, and 59, with total rates of 10.8%, 9.1%, 6.5%, and 6.2%, respectively. Similar results were observed in other studies.^18^, ^19^ The prevalence of HPV genotypes varied across regions. In China, the four most common HR‐HPV genotypes detected were HPV52, 16, 58, and 56, in descending order.

Notably, this study had certain limitations. First, in this study, the genotypes of the discordance sample should have been detected with a third (or more) kit(s) to accurately confirm a sample's genotype. Moreover, additional HPV genotyping detection kits should be used to determine the accuracy of the established method for genotyping the remaining 12 HR genotypes. Second, when assessing HPV genotypes in all levels of cervical lesions, most of the samples collected in this study were from patients undergoing routine gynecological examinations. Only two samples were diagnosed with HSIL, which is an insufficient number to be considered representative. Therefore, the 50% HPV infection rate in HISL was different from that in other studies which show significantly higher HPV infection rates in the HSIL population.^20^, ^21^

In summary, this study successfully established a detection method that can simultaneously detect 16 HPV genotypes. The method has good accuracy, specificity, sensitivity, and repeatability and can be achieved in a closed tube operation. For clinical HPV typing detection, this method can be extended to the accurate screening of pre‐vaccine samples in vaccine clinical trials and has the potential for broad application.

## FUNDING INFORMATION

The study was funded by the major project of Study on Pathogenesis and Epidemic Prevention Technology System by the Ministry of Science and Technology of China [grant number2021YFC2302500], the National Science and Technology Major Projects of Drug Discovery [grant number 2018ZX09101‐001], and by National Key R&D Program of China [grant number 2020AAA0105200].

## CONFLICT OF INTEREST STATEMENT

All authors have read and agreed to the published version of the manuscript. None of the authors have a conflict of interest to disclose.

## INSTITUTIONAL REVIEW BOARD STATEMENT

The study was conducted in accordance with the Declaration of Helsinki, and the protocol was approved by the Ethics Committee of Peking University First Hospital (2019 Instrument Registration No. 36, approved 12/31/2019).

## INFORMED CONSENT

Informed consent was obtained from all subjects involved in the study.

## Data Availability

All data supporting the findings of this study are available with the corresponding authors. The data are not publicly available, since it could compromise the privacy of the research participants.
